# Protocol for targeted gene manipulation and thermogenic evaluation in mouse brown adipocytes

**DOI:** 10.1016/j.xpro.2025.104317

**Published:** 2025-12-29

**Authors:** Jakub Bunk, Drishti Soni, Matthias Calderon, Bozena Samborska, Lawrence Kazak

**Affiliations:** 1 Rosalind & Morris Goodman Cancer Institute, McGill University, Montreal, QC H3A 1A3, Canada; 2 Department of Biochemistry, McGill University, Montreal, QC H3G 1Y6, Canada

**Keywords:** genetics, metabolism, molecular biology, gene expression

## Abstract

Adeno-associated viruses (AAVs) are versatile, non-integrating vectors for *in vivo* gene delivery. We present a reproducible workflow for generating Cre-dependent FLEX-AAVs, quantifying viral titer, and performing localized injections for cell-type-specific transgene expression in mice. The protocol also details the assessment of thermogenic capacity in genetically modified brown adipocytes using Clark-type electrode respirometry.

For complete details on the use and execution of this protocol, please refer to Bunk et al.^1^

## Highlights

• Instructions for cloning of a gene of interest into a FLEX cassette
• Procedures for high-titer AAV preparation in HEK293T/17 cells
• Guidelines for surgical AAV injection into interscapular brown adipose tissue
• Steps for assessing thermogenesis of modified adipocytes using respirometry

## Abstract (editor highlights)

Publisher’s note: Undertaking any experimental protocol requires adherence to local institutional guidelines for laboratory safety and ethics.

## Graphical abstract

**Figure:**
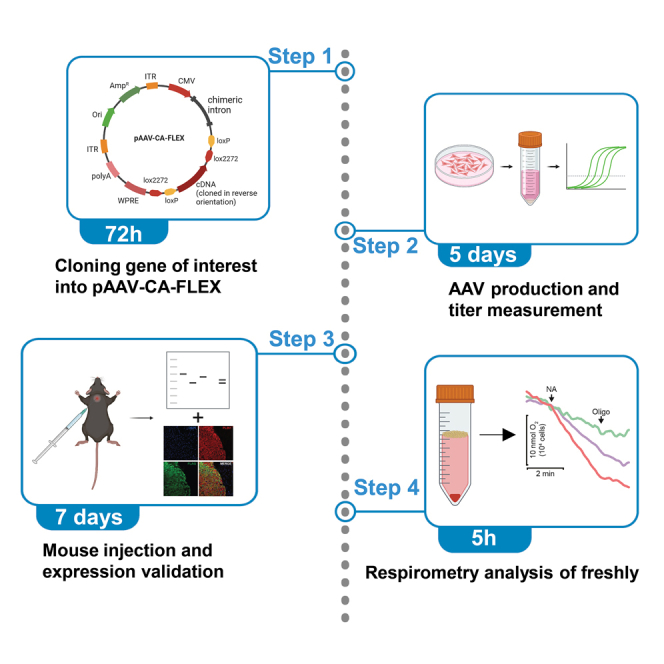

## Before you begin

Adeno-associated viruses (AAVs) are small, single-stranded DNA vectors widely used for in vivo gene delivery.^2^ Because AAVs remain episomal and do not integrate into the host genome, they provide a low-toxicity platform for long-term transgene expression.^3^ By combining Cre-dependent FLEX cassettes with localized injections, gene delivery can be achieved with both cell-type and anatomical specificity. This protocol describes a reproducible workflow for cloning, viral production, and functional analysis of thermogenic gene expression in brown adipose tissue. The gene of interest, illustrated here with the coding sequence (CDS) of green fluorescent protein (GFP), is cloned into a FLEX cassette, and high-titer AAV is produced and purified in HEK293T/17 cells. Viral genome copies are quantified by qPCR to ensure accurate dosing. The virus is injected into interscapular brown adipose tissue (iBAT) of Adiponectin-Cre mice to drive adipocyte-specific transgene expression *in vivo*.^1^ Finally, the thermogenic capacity of isolated brown adipocytes is assessed using a Clark-type electrode to measure oxygen consumption. Although GFP is used here as an example, the workflow can be readily adapted for adipocyte-specific restoration of thermogenic effectors to evaluate their functional contribution.

### Innovation

Gene manipulation in adipose tissue has traditionally relied on systemic viral delivery or germline transgenics. Although effective, these methods lack spatial precision, often produce off-target expression, and require high viral doses or complex breeding schemes that limit flexibility. Functional assessment of thermogenesis has similarly depended on whole-animal physiology or in vitro assays, with few approaches directly linking targeted in vivo gene manipulation to quantitative thermogenic output. Here, we describe a workflow for depot-restricted, Cre-dependent AAV delivery that enables precise gene expression in adipocytes. Combined with adipocyte isolation and functional respirometry, this method establishes a direct connection between genetic manipulation and thermogenic capacity. We focus on the futile creatine cycle (FCC), a UCP1-independent thermogenic pathway driven by creatine kinase B (CKB) and tissue-nonspecific alkaline phosphatase (TNAP),^4^^,^^5^^,^^6^^,^^7^^,^^8^^,^^9^^,^^10^^,^^11^^,^^12^^,^^13^ and demonstrate that this pipeline can delineate UCP1-independent thermogenesis through the FCC.^1^ This depot-specific genetic rescue approach, combined with precise subcellular targeting, can be broadly applied to dissect the contribution of other futile substrate cycles to energy expenditure. Moreover, the method described herein has broader applicability as it could be applied to gene expression silencing and also applied to influence gene expression in other cell types by changing the cre model.

### Institutional permissions

Mouse experiments were performed according to procedures approved under an Animal Use Protocol at McGill University, in compliance with guidelines set by the Canadian Council of Animal Care.

### Plasmid preparation

**Timing: 72 h**

1. Transform DH5α *E. coli* with pAAV-CA-FLEX.
2. Grow colonies at 37°C in selective medium with shaking for 8–12 h.
3. Harvest bacteria and purify plasmid DNA using a midi- or maxi-prep kit.
4. Confirm plasmid integrity by whole-plasmid sequencing (e.g., Plasmidsaurus).

### HEK29T cell preparation

**Timing: <1 week**

5. Seed HEK293T/17 cells in a 15 cm cell culture dish with DMEM containing 10% FBS and 1% Penicillin-Streptomycin.
6. Let the cells reach 90%–100% confluency, changing media every 48 h.
7. Polyethylenimine (PEI 25K) stock preparation: Dissolve PEI (1 mg/mL) in 60°C water, aliquot and store at −80°C until usage.

## Key resources table

**Table:** 

REAGENT or RESOURCE	SOURCE	IDENTIFIER
**Antibodies**		

VCL, Dilution 1:5,000	Cell Signaling Technology	E1E9V
PLIN1 (WB 1:1,000 and Immunofluorescence 1:200)	Cell Signaling Technology	9349
FLAG (WB), Dilution 1:2,000	Cell Signaling Technology	2368
FLAG M2 (Immunofluorescence), Dilution 1:1,000	MilliporeSigma	F1804
Anti-rabbit, Dilution 1:10,000	Promega	W4011
Antirabbit AlexaFluor-488, Dilution 1:200	Thermo Fisher Scientific	A11008
Anti-mouse AlexaFluor-594, Dilution 1:200	Thermo Fisher Scientific	A11005

**Bacterial and virus strains**		

DH5α	New England Biolabs (NEB)	C2987

**Chemicals, peptides, and recombinant proteins**		

PfuTurbo DNA Polymerase	Agilent	600250
Deoxynucleotide (dNTP) Solution Mix	New England Biolabs (NEB)	N0447S
HindIII-HF	New England Biolabs (NEB)	R3104S
SalI-HF	New England Biolabs (NEB)	R3138S
10× rCutSmart Buffer	New England Biolabs (NEB)	B6004S
Nuclease-free H_2_O –Dnase, RNase, Protease Free	Wisent Bioproducts	809-115-LL
Gel Loading Dye, Purple (6×)	New England Biolabs (NEB)	B7024S
FroggaStain, Nucleic acid stain	FroggaBio	FBSTAIN
Froggarose LE	FroggaBio	A87-500G
Instant Sticky-end Ligase Master Mix	New England Biolabs (NEB)	M0370L
Ampicillin	Bioshop	AMP201.5
DMEM, with Glutamine and Na-pyr	Wisent Inc.	319-005-CL
Fetal Bovine Serum	Wisent Inc.	090–105
Pen-strep 100×	Wisent Inc.	450-201-EL
Opti-MEM	Gibco	31985062
Polyethylenimine (PEI)	Polyscience Inc.	23966–100
Stericup Quick Release-HV Sterile Vacuum Filtration System, 0.45 μm pore size	Sigma-Aldrich	S2HVU02RE
5× AAVanced concentration reagent	System Biosciences (SBI)	AAV110A-1
PBS, 1×, pH 7.4, without calcium and magnesium	Wisent Inc.	311-010-CL
EcoRV-HF	New England Biolabs (NEB)	R3195S
DNase I, RNase-free (1 U/μL) Kit	Thermo Scientific	EN0521
DNA/RNA Extraction Reagent - ViralXpress	Sigma-Aldrich	3095
2-Propanol (Certified ACS)	Fisher Chemical	A416P-4
Ethyl Alcohol Anhydrous, USP	Greenfield Global	P016EAAN
2× SybrGreen	Promega	A6002
Isoflurane USP	Fresenius Kabi	CP0406V2
Carprofen	Zoetis	02255693
Saline Solution, 0.9%	Wisent Inc.	809089-CL
SYSTANE OINTMENT - Eye ointment	Alcon	2444062
BAXEDIN 2% CHG w/v with Isopropyl Alcohol 70% v/v, Untinted Solution	Omega Laboratories	UCASOL-L0000017
10 μL Model 901 Removable Needle (RN) Syringe, 26s gauge, 2 in, point style 2	Hamilton	CAL8370
Lidocaine	Teligent	02422026
Vetbond Tissue Adhesives	3M	1469SB
NaCl	Sigma-Aldrich	S5886
KCl	Sigma-Aldrich	P3911
CaCl_2_	Sigma-Aldrich	223506
MgCl_2_	BioShop	69-65-8
K_2_HPO_4_	Sigma-Aldrich	60353
NaHCO_3_,	Sigma-Aldrich	S5761
HEPES	Sigma-Aldrich	H4034
EDTA	BioShop	EDT001
Glucose	Sigma-Aldrich	G7528
Fatty acid-free BSA	Wisent Inc.	800-095-EG
Collagenase B	Worthington	LS004147
Soybean trypsin inhibitor	Worthington	LS003571
L-(−)-Norepinephrine (+)-bitartrate salt monohydrate	Sigma-Aldrich	A9512
Oligomycin	Sigma-Aldrich	495455
SBI-425	MedChem Express	HY-125756
carbonyl cyanide 3-chlorophenylhydrazone (CCCP)	Sigma-Aldrich	215911

**Critical commercial assays**		

QIAGEN Plasmid Mini Kit	QIAGEN	12123
QIAGEN Plasmid Midi Kit	QIAGEN	12143
QIAquick Gel Extraction Kit	QIAGEN	28706

**Experimental models: Cell lines**		

HEK293T/17	ATCC	CRL-11268

**Experimental models: Organisms/strains**		

B6.FVB-Tg(Adipoq-cre)1Evdr/J	The Jackson Laboratory	028020

**Oligonucleotides**		

Cloning FP-5′ cgtattgAAGCTTTCActtgtcat cgtcgtccttgtaatcctgagaaccaggtgcggtgtca gacttgtacagctcgtccatgccgagagtgatcccgg 3′	IDT	–
Cloning RP-5′ cgtattgGTCGACatggtgagcaagggcgagg 3′	IDT	–
qPCR FP-5′ cgctgctttaatgcctttgtat 3′	IDT	–
qPCR RP-5′ gggccacaactcctcataaa 3′	IDT	–

**Recombinant DNA**		

pAAV-CA-FLEX	Addgene	38042
AAV GFP (plasmid)	Addgene	49055
Plasmid - pDP8.ape	PlasmidFactory	PF0478

## Step-by-step method details

### Cloning GFP-FLAG into pAAV-CA-FLEX

**Timing: Approximately 72 h**

This section describes cloning a gene of interest into the FLEX cassette. Here, we use C-terminally FLAG-tagged green fluorescent protein (GFP-FLAG) as an example.

**CRITICAL:** The cDNA must be inserted in reverse orientation for Cre-dependent activation.

1. **Digest plasmid:** Linearize 1 μg of pAAV-CA-FLEX with HINDIII-HF and SalI-HF in a 50 μL reaction:

**Table:** Pipette Plan: Plasmid backbone linearization

Reagent	Final concentration	Amount
pAAV-CA-FLEX plasmid	20 ng/μL	**X** μL (1 **μ**g total)
10× rCutSmart Buffer	1×	5 μL
HindIII-HF	0.8 U/μL	2 μL
SalI-HF	0.8 U/μL	2 μL
Nuclease-free H_2_O	N/A	Up to 50 μL

**Figure 1:**
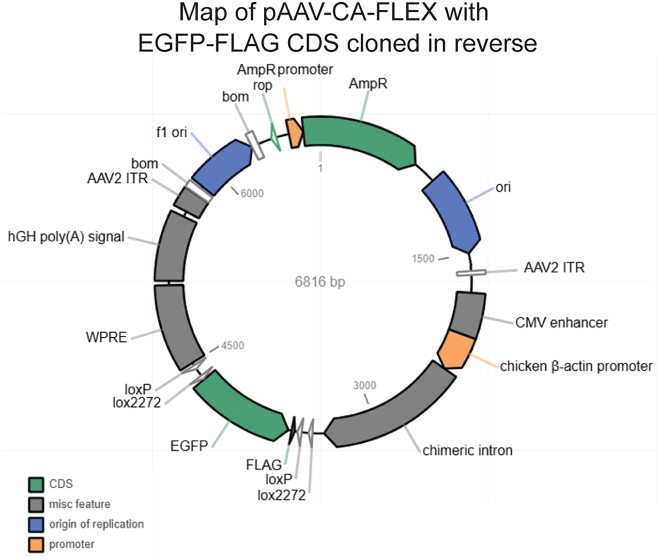
Map of pAAV-CA-FLEX with EGFP-FLAG CDS cloned in reverse orientation Sequencing was obtained with Plasmidsaurus.

2. Incubate at 37°C for 1 h.
3. **Verify digestion:** Add 10 μL 6× loading dye and run on 1% agarose gel.
4. Extract DNA using QIAquick Gel Extraction Kit, following the manufacturer’s protocol.

***Note:*** Run three control digests (uncut, single cuts of each enzyme) to confirm complete digestion.

***Note:*** Undigested and linearized pAAV-CA-FLEX migrate similarly.

5. **Amplify insert:** PCR-amplify GFP-FLAG with primers containing HindIII and SalI sites (Table 1).

**Table 1:** PCR reaction conditions of insert

Steps	Temperature	Time (min)	Cycles
Initial Denaturation	98°C	3:00	1
Denaturation	98°C	0:30	–
Annealing	65°C	0:15	39 cycles
Extension	72°C	2:00	–
Final Extensions	72°C	5:00	1
Store at	4°C	∞	N/A

**Table:** Pipette Plan: Cloning PCR reaction mix

Reagent	Final concentration	Amount
AAV GFP (plasmid)	–	**X** μL (∼10 ng total)
Primer_Forward (10 μM stock) cgtattgAAGCTTTC Acttgtcatcgtcgtccttgtaatcctgagaaccaggtgcggtgt cagacttgtacagctcgtccatgccgagagtgatcccgg	0.5 μM	2.5 μL
Primer_Reverse (10 μM stock) cgtattgGTCGACatg gtgagcaagggcgagg	0.5 μM	2.5 μL
dNTP mix (10 mM each)	0.2 mM each	1 μL
Pfu DNA polymerase 10× Buffer with MgSO_4_	1×	5 μL
Pfu TURBO High Fidelity DNA polymerase	0.05U/μL	1 μL
Nuclease-free H_2_O	N/A	Up to 50 μL

6. Add 10 μL 6× loading dye and run on 1% agarose gel.
7. Extract DNA using QIAquick Gel Extraction Kit, following the manufacturer’s protocol.
8. Elute DNA in 35 μL of Nuclease-free H_2_O.
9. Digest insert: Repeat digestions as above using HindIII-HF and SalI-HF. Run on gel and extract DNA. Digest 30 μL of the gel-extracted insert with HindIII-HF and SalI-HF in a 50 μL reaction:

**Table:** Pipette Plan: Digestion of PCR-amplified insert

Reagent	Final concentration	Amount
PCR-amplified insert (i.e., GFP-FLAG)	N/A	30 μL
10× rCutsmart buffer	1×X	5 μL
HindIII-HF	0.8 U/μL	2 μL
SalI-HF	0.8 U/μL	2 μL
Nuclease-free H_2_O	N/A	Up to 50 μL

10. Incubate at 37°C for 1 h.
11. Add 10 μL of 6× Loading Dye and run the product of the digestion on a 1% agarose gel.
12. Excise and extract the DNA as mentioned in step 7.
13. Set up a ligation reaction with a 1:3 molar ratio of digested plasmid backbone:GFP–Flag insert by mixing the following components and incubate for 1 h at 20°C–25°C.

**Table:** Pipette Plan: Ligation of insert into pAAV-CA-FLEX

Reagent	Final concentration	Amount
Linearized pAAV-CA-FLEX (plasmid backbone DNA)	0.5–2 fmol/μL	5-20 fmol
Insert	1.5–6 fmol/μL	3× molar excess (15-60 fmol)
Nuclease-free H_2_O	N/A	Up to 5 μL
Instant Sticky-end Ligase (2×)	1×	5 μL

14. Mix gently by flicking, and incubate 60 min at 20°C–25°C.
15. Transform 3 μL of ligation mix into DH5α competent cells.
16. Plate on LB-agar (supplemented with ampicillin).
17. Incubate plates upside down overnight at 37°C.
18. Grow colonies in 5 mL LB (supplemented with ampicillin) overnight at 37°C.
19. Isolate plasmid DNA (pAAV-CA-FLEX with insert of choice) and sequence to confirm insertion in reverse orientation (Figure 1).

### HEK293T/17 cell culture, transfection, AAV production, and AAV harvest

**Timing: Approximately 5 days**

This section describes producing high-titer AAV in HEK293T/17 cells.

20. Transfect HEK293T/17 cells:
  **CRITICAL:** At the time of transfection, cells must be ≥90% confluent.
  a. Seed HEK293T/17 cells in 15-cm dishes with DMEM + 10% FBS + 1% Pen/Strep and keep in humidified incubator at 37°C and 5% CO_2_.
  b. Three hours before transfection, replace medium with 25 mL DMEM containing 5% FBS + 1% Pen/Strep.
  c. Prepare transfection cocktail in a tube by mixing the following:

**Table:** Pipette plan: HEK293T/17 cell transfection

Reagent	Final concentration	Amount (for one 15 cm dish)
OptiMEM	N/A	2.5 mL
pAAV-CA-FLEX_GFP-FLAG	3.7 μg/mL	10 μg
pDP8.ape (AAV8)	14.6 μg/mL	39.5 μg
PEI	74.1 μg/mL	200 μL
    **CRITICAL:** Avoid freeze-thawing of PEI solution. Add PEI last to avoid DNA precipitation.
    ***Note:*** While we use AAV8 to transduce murine adipose tissue, other groups have reported that other serotypes can be used.^14^
  d. Mix by inverting tube; incubate 10–15 min standing at 20°C–25°C.
  e. Gently add HEK293T/17 cell transfection mix dropwise to cells, return to incubator gently.
  f. After 24 h, replace with 20 mL DMEM (containing 10% FBS + 1% Pen/Strep).
    ***Note:*** The 20 mL media volume is important for subsequent steps.
  g. Keep cells in humidified incubator at 37°C and 5% CO_2_ for 48 h.
21. Harvest cells:
  a. Scrape cells and collect them together with the media they were cultured in. One 50 mL tube is used for cells and media from two 15-cm dishes.
  b. Centrifuge 15 min at 3,000 × *g*, 4°C to pellet cell debris.
  c. Filter the supernatant (media) through a 0.45 μm Stericup filter.
  d. Aliquot 40 mL of filtered media into clean 50 mL tubes.
  e. Add 10 mL of pre-chilled 5× AAVanced concentration reagent to every 40 mL of filtered media. Invert five times to mix. Incubate minimum of 24 h at 4°C (do not exceed five days of incubation).
  f. Centrifuge 30 min at 1,500 × *g*, 4°C.
  g. Viral particles will appear as a whitish pellet (Figures 2A and 2B).
  h. Resuspend pellet in 300–400 μL of sterile PBS.
  i. Aliquot virus suspension into small volumes (the volume will depend on needs of the user).
  j. Flash-freeze aliquots in liquid nitrogen and store at −80°C.

**CRITICAL:** Save two-to-three 5 μL aliquots in separate 1.5 mL tubes for future titration. As you improve in the method, less tubes can be saved for titering.

**Figure 2:**
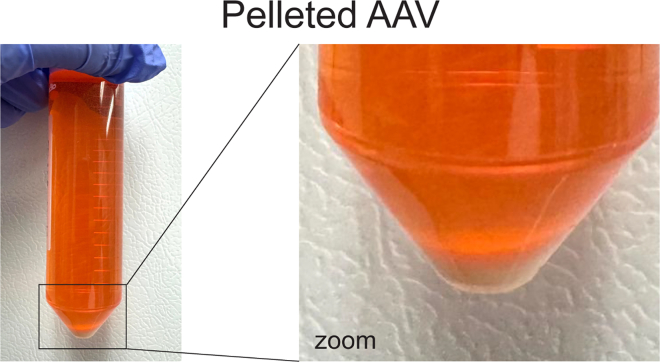
Photo of pelleted AAV

### AAV titering by qPCR

**Timing: 5–6 h**

This section describes a qPCR-based method to quantify AAV particles.

**CRITICAL:** Prepare a fresh linearized plasmid standard each time. You can store at 4°C on the day of extraction for 3 h maximum before use.

22. Preparation of the standard curve:
  a. Linearize the pAAV-CA-FLEX plasmid with EcoRV-HF. Mix the following components and incubate for 1 h at 37°C.

**Table:** Pipette plan: Backbone plasmid linearization for titering standard curve

Reagent	Final concentration	Amount
pAAV-CA-FLEX plasmid	0.06 μg/μL	**X** μL (3 μg total)
10× rCutsmart buffer	1×	5 μL
EcoRV-HF	0.8 U/μL	2 μL
Nuclease-free H_2_O	N/A	Up to 50 μL
  b. Add 10 μL of 6× Loading Dye into your digest and perform gel electrophoresis on a 1% agarose gel.
  c. Gel extract the linearized plasmid DNA (QIAquick Gel Extraction Kit).
  d. Measure DNA concentration (NanoDrop or equivalent).
  e. Calculate molecules of linearized plasmid/μL by using https://www.bioinformatics.org/sms2/dna_mw.html. Paste the entire plasmid sequence into the text area and calculate molecules of linearized plasmid/μL. Make sure to select “double” in the “Treat sequence as” box. Click Submit.
  f. Now that you have your molecules of linearized plasmid/μL, prepare a stock solution of 5 × 10^8^ molecules/μL of linearized plasmid, and then make 10-fold serial dilutions (5 × 10^8^, 5 × 10^7^, 5 × 10^6^, and 5 × 10^5^ molecules/μL).
    ***Note:*** Remember that for accurate titering the TOTAL molecules of linearized plasmid per well will need to be used. For example, if you load 2 μL of 5 × 10^8^/μL, this is equivalent to 10^9^ molecules per well, which is a log of 9.
23. Preparation of AAV samples to titrate against the standard curve:
  a. Remove ambient non-viral DNA from the sample via DnaseI treatment.
    ***Note:*** This step ensures that all DNA not protected by viral capsid is removed from the sample before the actual AAV is titered.

**Table:** Pipette plan: DNaseI treatment (50 μL volume)

Reagent	Final concentration	Amount
Viral sample	N/A	5 μL
10× DNase buffer	1×	5 μL
DNaseI	0.02U/μL	1 μL
Nuclease-free H_2_O	N/A	39 μL
  b. Mix gently by flicking tube.
  c. Incubate 30 min at 37°C.
  d. Heat-inactivate DNaseI at 75°C for 10 min; quick spin (∼5 seconds) max speed.
  e. Add 200 μL ViralXpress reagent; vortex 10 seconds.
  f. Incubate 7.5 min at 20°C–25°C; quick spin (∼5 seconds) max speed.
  g. Add 250 μL isopropanol; vortex 10 seconds; centrifuge at 16,000 × *g* for 10 min at 20°C–25°C.
  h. Wash pellet with 800 μL 70% ethanol; centrifuge at 16,000 × *g* for 10 min at 20°C–25°C.
  i. Remove residual supernatant; air-dry pellet ≤5 min in fume hood.
  j. Resuspend pellet in 50 μL nuclease-free water; dissolve at 55°C.
  k. Prepare dilutions (1:10, 1:100, 1:1,000) for qPCR for viral titration (Table 2).
    ***Note:*** Resuspending in 50 μL (step 2i) yields a 1:10 viral DNA suspension.
    ***Note:*** Avoid drying the DNA pellet for more than 5 min.

**Table:** Pipette plan: Titering qPCR reaction mix (10 μL volume)

Reagent	Final concentration	Amount
DNA template (viral or standard)	N/A	2 μL
2× SybrGreen	1×	5 μL
2.5 μM Primer Forward 5′ – cgctgctttaatgcctttgtat – 3′	0.25 μM	1 μL
2.5 μM Primer Reverse 5′ – gggccacaactcctcataaa – 3′	0.25 μM	1 μL
Nuclease-free H_2_O	N/A	1 μL

**Table 2:** Titering qPCR reaction parameters

Steps	Temperature	Time (min)	Cycles
Initial Denaturation	95°C	3:00	1
Denaturation	95°C	0:05	–
Annealing	60°C	0:25	39
Extension	72°C	0:20	–
	*95°C*	*0:10*	*1*
Melt Curve Determination	*65°C*	*0:31*	*1*
	*65°C + 0.5°C/cycle*	*0:05*	*60*

**Note:** 10 μL volume, Lid: 105°C.

**Note:** Load technical duplicates of each sample (standard or viral DNA).
  l. Calculate the qPCR reaction efficiency (should be > 80%). Plot the Cq values (Figure 3A) of each standard dilution on the y axis and the Log value of molecules of linearized plasmid (see **Note** in Step 3.1.f) on the x axis (Figure 3B). Calculate the qPCR efficiency using the equation provided in Figure 3C.
  m. Use the slope equation of the standard curve (Figure 3B) to calculate the log value of the viral molecules of your sample per well (x = $\frac{y-b}{m}$), where y = the Cq of the viral sample; m (the slope) and b (the y-intercept) are defined from the standard curve equation (Figure 3B). The viral particles per well of each dilution will be expressed as 10^*x*^.
  n. To obtain the viral particles per well (2 μL of sample) multiply 10^*x*^ by the corresponding dilution factor, take the average, and then multiply by 500× to obtain viral particles per mL.

**Figure 3:**
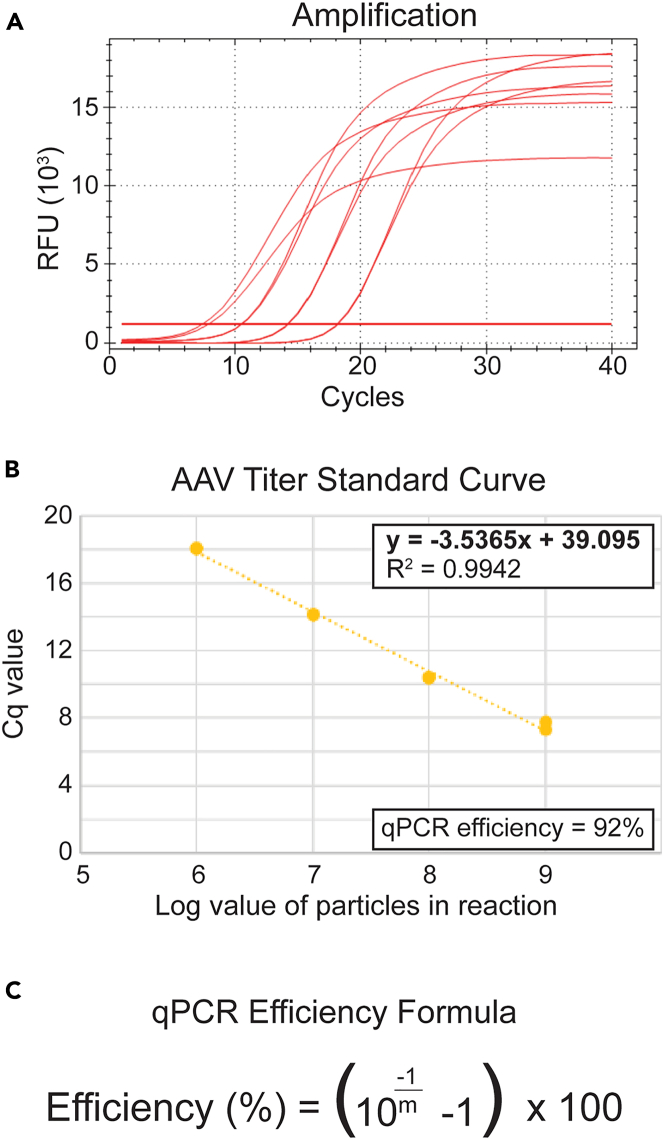
The results of titering qPCR for standard curve (A) The fluorescence signal from SybrGreen of standard curve dilutions. (B) Obtaining the slope equation and qPCR efficiency of standard curve. (C) qPCR efficiency formula (https://toptipbio.com/primer-efficiency-calculator/).

### Surgically directed AAV injection

**Timing: ∼30 min per mouse for injection. Wait 7 days minimum to allow recovery from surgery**

This section describes localized delivery of Cre recombinase-dependent AAV into interscapular brown adipose tissue (iBAT).

**CRITICAL:** Keep the incision small (∼0.5 cm) to enable incision closure with tissue glue rather than stitches.

***Note:*** Prior to starting surgery, inject mice subcutaneously with 0.5 mL of saline (for hydration) and 0.02 mg/g body weight of carprofen (equivalent to an injection volume of 5 μL/g body weight using a 4 mg/mL stock).

24. Pre-Surgical Preparation.
  a. Dilute AAV in sterile saline to the desired viral concentration of viral particles per 20 μL (injection volume); keep on ice.
    ***Note:*** We typically aim for 1–1.5 × 10^11^ viral particles/mouse.
  b. Anesthetize mice with isoflurane (2.5% in 0.8 l/min oxygen).
  c. Apply ophthalmic ointment to prevent corneal drying.
  d. Shave fur above iBAT and disinfect with BAXEDIN (2% w/v chlorhexidine gluconate in 70% v/v isopropyl alcohol).
  e. Confirm deep anesthesia using a toe pinch.
25. Viral Injection (see Methods video S1).
  a. Make a ∼0.5 cm incision above the iBAT, parallel to the spine.
  b. Gently separate iBAT from surrounding tissue by blunt dissection.
  c. Hold the iBAT lobe with fine forceps.
  d. Inject 2.5 μL of viral dilution at four sites per lobe (10 μL/lobe = 20 μL/mouse).
26. Wound Closure and Recovery.
  a. Apply a drop of lidocaine locally at the incision site.
  b. Close incision with surgical tissue adhesive (Vetbond).
  c. Provide carprofen (0.02 mg/g body weight) once daily for 2 days post-surgery and monitor animals daily for one week.

Methods video S1. Surgically directed injection of AAV into iBAT, related to Step 25

### Respirometry analysis of freshly isolated brown adipocytes

**Timing: ∼5 h**

This section describes a methodology for isolating mature brown adipocytes from interscapular brown fat and their respirometry assessment using Clark-type electrode (Rank Brothers).

**CRITICAL:** Brown adipocytes should be freshly isolated and gently handled to avoid cell rupture.

27. Prepare Digestion buffer:

**Figure 4:**
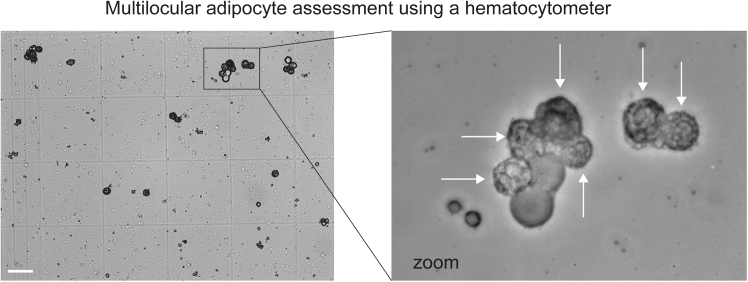
Image of isolated brown adipocytes Scale bar, 100 μm.

**Figure 5:**
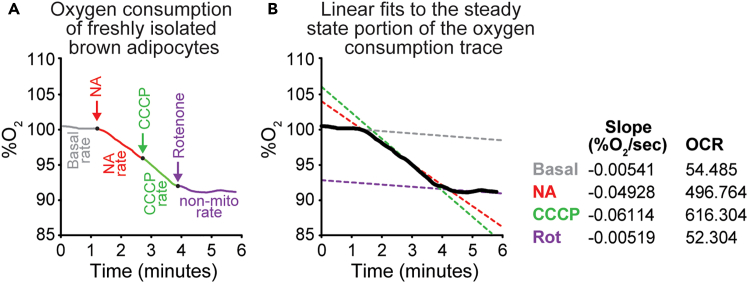
Oxygen Consumption Rate obtained from wild-type adipocytes (A) Oxygen consumption trace from 10,000 freshly isolated brown adipocytes from 10-12-week-old wild-type mice. Sequential additions of noradrenaline (NA, 0.1 μM), carbonyl cyanide 3-chlorophenylhydrazone (CCCP, 40 μM), and rotenone (Rot, 5 μM) are indicated by arrows. Colored segments highlight the oxygen consumption response to each compound. (B) Linear fits to the steady-state portion of each trace following compound addition were used to calculate oxygen consumption rates (OCR) per million cells per minute (shown in the table to the right).

Recipe: Krebs-Ringer bicarbonate buffer

***Note:*** KRBMB is a component of the Digestion buffer and can be prepared ahead of time and stored at 4°C for up to a month.

**Table:** 

KRBMB	Final concentration
NaCl	135 mM
KCl	5 mM
CaCl_2_	1 mM
MgCl_2_	1 mM
K_2_HPO_4_	0.4 mM
NaHPO_4_	25 mM
HEPES	20 mM

Recipe: Digestion buffer

***Note:*** Prepare and use on the day of experiment.

**Table:** 

Digestion buffer	Final concentration	For 5 mice digest
KRBMB	N/A	8 mL
Collagenase B	2 mg/mL	20 mg
Soybean Trypsin Inhibitor	1 mg/mL	10 mg
D-Glucose	10 mM	18 mg
20% Fatty acid-free BSA diluted in water (0.22 μm-filtered)	4%	2 mL

28. Brown adipocyte isolation (this protocol uses 5 mice):
  a. Euthanize mice by direct cervical dislocation and excise interscapular brown adipose tissue (iBAT). Keep iBAT immersed in cold PBS until all samples are collected.
  b. Blot excess PBS with a Kimtech wipe to dry pooled iBAT tissue.
  c. Transfer tissues into a 10 mL glass beaker and finely mince using spring scissors for ∼7 min.
  d. Transfer minced tissue into 10 mL of digestion buffer in a 50 mL conical tube.
  e. Shake at 150 rpm in a 37°C water bath for 50 min. Vortex for 2 s every 5 min during digestion.
  f. Pass the digested suspension through a 100 μm cell strainer into a 50 mL tube containing 10 mL stop buffer (4 mM EDTA, pH 8.0; 3% BSA in PBS).
  g. Gently invert the tube three times and incubate upright at 20–25°C for 40 min to allow adipocytes to float.
  h. Using a plastic Pasteur pipette, collect the fat layer and transfer it into a fresh 15 mL tube containing 10 mL DMEM/F12 GlutaMAX + 10% FBS.
    ***Note:*** Aspirate slowly. Avoid transferring clumped white fat.
  i. Invert the 15 mL tube gently three times and incubate upright for 40 min at 20°C–25°C to allow adipocytes to float again.
  j. Collect the brown adipocyte layer and transfer into a 5 mL tube containing 2 mL fresh DMEM/F12 GlutaMAX + 10% FBS.
29. Assessment of brown adipocyte number:
  a. Gently invert the 5 mL tube three times to distribute cells evenly.
  b. Using a wide-bore pipette tip, load 10 μL of adipocyte suspension into a Bright-Line Hemocytometer (Hausser Scientific).
  c. Count only multilocular adipocytes (see white arrows in Figure 4).

**CRITICAL:** If concentration exceeds 300,000 adipocytes/mL, dilute the sample and recount.

***Note:*** Viability stains may be used to distinguish live cells from dead cells; however, in our experience, dyes like trypan blue are hard to detect due to the small cytoplasmic area because of lipid droplets.

30. Measuring oxygen consumption of brown adipocytes: *Set up and calibrate the Clark-type electrode (Rank Brothers) as previously described*.^15^
  a. Add DMEM/F12 + 10% FBS to the electrode chamber.
  b. Allow equilibration with atmospheric oxygen until a stable signal is obtained.
    ***Note:*** Rate at this stage reflects electrode drift.
    ***Note:*** Electrode drift should not exceed 1% O_2_ per 5 min.
    ***Note:*** Excessive noise indicates improper setup and requires electrode reassembly.
  c. Add 10,000 brown adipocytes to the chamber (final volume = 0.7 mL, 37°C).
  d. Seal the chamber with a plunger.
  e. Record basal respiration (prior to drug addition) for 30–60 s once the trace stabilizes.
    **CRITICAL:** Use only the linear portion of the basal trace.
  f. Add thermogenic activators or inhibitors via a Hamilton syringe to the stirring chamber.
    ***Note:*** A transient change in O_2_% may occur upon needle insertion; allow 15–30 s for stabilization.
  g. Ensure that different treatment groups are measured using the same electrodes to control for electrode-specific variability.
  h. Acquire data using the Rank Brothers Dual Digital Model 20 with PicoLog 6 software.
31. Data analysis:
  ***Note:*** Quantify OCR using the linear region of each trace (see Figure 5).
  a. Export data as “%O_2_ vs time (s)” from PicoLog 6 (.csv format).
  b. Import into OriginPro 2025 (or GraphPad Prism) for best-fit line analysis.
  c. Determine slope values representing rate of O_2_ consumption (%O_2_ s^-1^).
    Example Parameters.
    i. *Electrode Drift:* before cell addition (stabilized electrode).
    ii. *Basal Respiration:* post-cell addition, before drugs.
    iii. *Drug-driven Respiration:* after compound addition.
  d. Calculate oxygen consumption rate (OCR) per 10^6^ cells using:
    $$OCR\;\left(nmoles\;O_{2}\;\min^{-1}\cdot 10^{-6}cells\right)=-m\times 2.4\times V\times 60\times \frac{1,000,000}{c}$$
    ***m*:** rate of O_2_ consumption (%O_2_ s^-1^).
    ***V*:** total reaction volume (mL) in chamber.
    ***c*:** number of brown adipocytes in chamber.

## Expected outcomes

**Figure 6:**
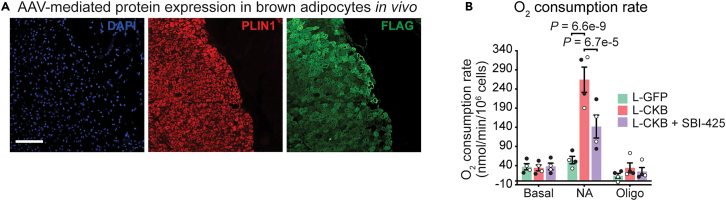
Assessment of Futile Creatine Cycle within mature brown adipocytes (A) Representative immunofluorescence images of iBAT transduced with AAV-FLEX-GFP-FLAG. Mature adipocytes were labelled with anti-Perilipin 1 (PLIN1) antibody (red), GFP-FLAG was labeled with anti-FLAG antibody (green). Nuclei were labelled with DAPI (blue). Scale bar, 100 μm. Figure adapted from figure 1d of Bunk et al.^1^ (B) Oxygen consumption rates basally and following sequential additions of noradrenaline (NA, 0.1 μM) and oligomycin (Oligo, 15 μM). SBI-425 (10 μM) was added at the start (n = 2 independent preparations/sex). Figure adapted from figure 5b of Bunk et al.^1^ Two-way ANOVA (Tukey’s post-hoc test). Data are represented as mean ± SEM.

This protocol provides a framework to achieve anatomically precise, cell-type specific expression of a gene of interest. HEK293T cells should exhibit somewhat round morphology at the time of AAV harvest but be attached to the bottom of the culture dish throughout the process. Viral titer should be roughly 1 × 10^13^, with the efficiency of qPCR reaction >80%. Injection of 1 × 10^11^ viral particles into cre recombinase-expressing adipocytes, should be sufficient for detection of protein of interest protein by western blot and immunofluorescence analysis selectively in cells positive for cre recombinase (Figure 6).

As emphasized in a recent perspective addressing ongoing debates in brown fat research,^16^ an agreed upon strategy to evaluate the contribution of a thermogenic pathway is to reintroduce its effector protein into a background lacking other major thermogenic mechanisms, thereby avoiding compensatory effects. Following this rationale, we reintroduced mitochondrial CKB into brown adipocytes of mice lacking endogenous *Ckb* and *Ucp1* using the FLEX-AAV system. This approach enabled us to directly assess the contribution of the FCC to thermogenesis in mature brown adipocytes (Figure 6).

## Limitations

One key limitation of AAV vectors is that they have a packaging limitation of ∼4.7 kb, constraining the size of insertable transgenes. Another limitation is the qPCR-based viral titration, which quantifies viral genomes rather than infectious particles. However, since DNaseI treatment is used during AAV titering, consistent gene expression is obtained across samples. When isolating AAV8 from a single 15 cm dish utilizing the method described here, the final titer we obtain is 0.5 × 10^13^ – 1 × 10^13^ viral particles/mL, when resuspended in 150–200 μL of sterile PBS. Lastly, surgical administration of AAV8 virus in this protocol could be invasive and therefore requires technical refinement.

## Troubleshooting

### Problem 1

Low viral titer.

### Potential solution

To avoid low viral titer make sure to use HEK293T/17 cells from an early passage. We recommend titrating the backbone-to-transfer plasmid ratio and PEI concentration. If the problem persists scale up the number of plates per preparation.

### Problem 2

HEK293T/17 cells detach during transfection.

### Potential solution

Make sure to apply the transfection mix gently to avoid cell lifting.

### Problem 3

Low qPCR efficiency.

### Potential solution

We recommend confirming the efficiency of primers, and designing alternative primers if needed. Optimize PCR conditions by changing the primer concentration or master mix composition.

### Problem 4

No protein overexpression.

### Potential solution

Verify protein expression using a control plasmid. Make sure that the cDNA is cloned in the correct orientation and the selected AAV serotype has efficient tropism for the target tissue.

### Problem 5

Nonspecific expression.

### Potential solution

Make sure there are no mutations in loxP sites, and confirm that the promoter driving the expression of cre recombinase is specific to the intended cell-type; if not, select an alternative cre-driver line.

## Resource availability

### Lead contact

Further information and requests for resources and reagents should be directed to and will be fulfilled by the lead contact, Lawrence Kazak (lawrence.kazak@mcgill.ca).

### Technical contact

For technical specifics on executing the protocol, Jakub Bunk (jakub.bunk@mail.mcgill.ca) and Lawrence Kazak (lawrence.kazak@mcgill.ca) will provide support to ensure its correct implementation.

### Materials availability

Please address requests for materials to Lawrence Kazak (lawrence.kazak@mcgill.ca).

### Data and code availability

• All data are available from the lead author upon request.
• This article does not report original code.
• Any additional information required to reanalyze the data reported in this article is available from the lead contact upon request.

## Acknowledgments

This work was supported by 10.13039/501100007202CIHR Project Grants (PJT-159529 and PJT-180557 to L.K.) and a Graduate Scholarship from the 10.13039/501100020951Fonds de Recherche de Quebec – Santé (FRQS) (to J.B.). L.K. is a Canada Research Chair in Adipocyte Biology.

## Author contributions

J.B. and L.K. conceived the project and designed the experiments. J.B., D.S., and M.C. developed and optimized the protocols. All authors contributed to manuscript writing/editing. L.K. supervised the work, acquired funding, and wrote the manuscript.

## Declaration of interests

The authors declare no competing interests.
